# Validity and reliability of the Fatigue Severity Scale in Finnish multiple sclerosis patients

**DOI:** 10.1002/brb3.743

**Published:** 2017-06-09

**Authors:** Eija Rosti‐Otajärvi, Päivi Hämäläinen, Anna Wiksten, Tanja Hakkarainen, Juhani Ruutiainen

**Affiliations:** ^1^ Department of Neurosciences and Rehabilitation Tampere University Hospital Tampere Finland; ^2^ Finnish Neuro Society Masku Finland; ^3^ StatFinn Oy Turku Finland; ^4^ Novartis Finland Oy Espoo Finland; ^5^ University of Turku Turku Finland

**Keywords:** fatigue, Fatigue Severity Scale, multiple sclerosis, reliability, self‐report, validity

## Abstract

**Background:**

Fatigue is one of the most debilitating symptoms in multiple sclerosis (MS) considerably interfering with patients’ daily functioning. Both researchers and clinicians need psychometrically robust methods to evaluate fatigue in MS.

**Objectives:**

The objective of this study was (i) to evaluate the psychometric properties of the Finnish version of the Fatigue Severity Scale (FSS) and (ii) to describe the results among patients with MS.

**Methods:**

In total, 553 patients with MS (mean age, 53.8 years; standard deviation [*SD*], 11.4; 79% women: mean patient‐defined disease severity, Expanded Disability Status Scale [EDSS] 4.0, *SD*, 2.5) completed the self‐administered questionnaires including the FSS. A standard procedure was used for the translation of the FSS.

**Results:**

The mean (*SD*) score for the FSS was 4.5 (1.7); in 65% of the patients, the score was ≥4.0. The data quality of the FSS was excellent, with 99.6% of computable scale scores. Floor and ceiling effects were minimal. The FSS showed high internal consistency (Cronbach's alpha, 0.95). Unidimensionality was supported based on confirmatory factor analysis with the comparative fit index being 0.94. The FSS showed moderate/high correlations with the perceived burden of the disease, quality of life and disease severity, whereas, age or gender did not have a significant effect on the FSS score.

**Conclusions:**

The Finnish version of the FSS showed satisfactory reliability and validity and thus can be regarded as a feasible measure of self‐reported fatigue.

## 1 INTRODUCTION

Fatigue is considered to be one of the most common and disabling symptoms of multiple sclerosis (MS), affecting about 80% of patients (Minden et al., 2006; Weiland et al., 2015). However, there is, no universally accepted definition for fatigue. MS‐related fatigue has been reported to manifest itself as an overwhelming sense of tiredness and lack of energy that affects a patient's participation in the activities of daily living and work. Fatigue is observed at all stages of disability and clinical forms of the disease (Induruwa, Constantinescu, & Gran, 2012). The causes of fatigue in MS are multifactorial and not well understood. Fatigue has been associated with dysfunction in the central nervous system and in immune‐ and neuroendocrine regulation. Pro‐inflammatory cytokines, over activity of neural circuits, defects in pre‐frontal basal ganglia circuitry, and axonal injury have been suggested as possible mechanisms (Induruwa et al., 2012). Depressive symptoms, impaired sleep, heat sensitivity, physical deconditioning, and medications have also been related to fatigue in MS (Induruwa et al., 2012).

Fatigue assessment typically relies on subjective self‐report questionnaires. Fatigue has been reported as a more frequent symptom in patients with higher disability (Amtmann et al., 2012; Armutlu et al., 2007; Mills & Young, 2010; Valko, Bassetti, Bloch, Held, & Baumann, 2008), in association with unemployment (Johansson, Ytterberg, Hillert, Widen, & von Koch, 2008; Mills & Young, 2010) as well as in progressive phenotypes of the disease (Mills & Young, 2010). Conversely, no significant association with demographic factors, such as age (Mills & Young, 2010; Valko et al., 2008) or gender (Valko et al., 2008) has been reported. A frequently used inventory for the evaluation of fatigue is the Fatigue Severity Scale (FSS) developed by Krupp et al. for the use in patients with systemic lupus erythematosus and MS (Krupp, LaRocca, Muir‐Nash, & Steinberg, 1989). The FSS, a nine‐item questionnaire, primarily focuses on the motor aspects of fatigue, the main emphasis being the assessment of the severity of fatigue symptom and its impact on an individual's daily functioning. Each item of the questionnaire is scored on a seven‐point Likert scale ranging from 1 (“completely disagree”) to 7 (“completely agree”; Table 1). The mean score of the nine items is used as the FSS score. Originally, the cut‐off score for fatigue was set to be ≥4 (Krupp et al., 1995), because fewer than 5% of healthy controls rated their fatigue above this level while 60%–90% of patients with medical disorders experienced fatigue at or above this level (Krupp et al., 1989). Subsequent studies have recommended the same cut‐off score (Armutlu et al., 2007; Valko et al., 2008). The categorization into non‐fatigue (FSS ≤4.0), borderline fatigue (4.0 < FSS < 5.0) and fatigue (FSS ≥5.0) has also been suggested (Johansson, Ytterberg, Back, Holmqvist, & von Koch, 2008; Ottonello, Pellicciari, Giordano, & Foti, 2016).

**Table 1:** FSS: English and Finnish versions

1.	My motivation is lower when I am fatigued./Olen haluttomampi mihinkään, kun olen uupunut.
2.	Exercise brings on my fatigue./Liikunta uuvuttaa minua.
3.	I am easily fatigued./Uuvun helposti.
4.	Fatigue interferes with my physical functioning./Uupumus haittaa fyysisiä toimintojani.
5.	Fatigue causes frequent problems to me./Uupumus aiheuttaa usein minulle ongelmia.
6.	My fatigue prevents sustained physical functioning./Uupumus estää pitempiaikaisen fyysisen toiminnan.
7.	Fatigue interferes with carrying out certain duties and responsibilities./Uupumus haittaa minua tiettyjä tehtäviä hoitaessani.
8.	Fatigue is among my three most disabling symptoms./Uupumus kuuluu kolmen eniten toimintakykyäni estävän oireen joukkoon.
9.	Fatigue interferes with my work, family, or social life./Uupumus haittaa työ‐ ja perhe‐elämääni tai ihmissuhteitteni hoitoa.

FSS, Fatigue Severity Scale.

Validation studies have been conducted with the English (Amtmann et al., 2012; Krupp et al., 1989), Arabic (Al‐Sobayel et al., 2016), Turkish (Armutlu et al., 2007), Swiss (Valko et al., 2008), Norwegian (Lerdal, Wahl, Rustoen, Hanestad, & Moum, 2005), German (Rietberg, van Wegen, & Kwakkel, 2010), Greek (Bakalidou, Skordilis, Giannopoulos, Stamboulis, & Voumvourakis, 2013), Italian (Ottonello et al., 2016), Portuguese (Laranjeira, 2012), and Persian (Fereshtehnejad et al., 2013) versions of the FSS. The assessments of the psychometric properties based on the classical test theory have shown moderate/high correlations between the FSS and other fatigue scales (Amtmann et al., 2012; Flachenecker et al., 2002; Krupp et al., 1989; Learmonth et al., 2013; Rietberg et al., 2010). Significant correlations between the FSS and other clinical and health‐related parameters, such as disease severity (Amtmann et al., 2012; Armutlu et al., 2007; Flachenecker et al., 2002; Valko et al., 2008), depression (Amtmann et al., 2012; Armutlu et al., 2007; Bakalidou et al., 2013; Flachenecker et al., 2002), pain (Amtmann et al., 2012), and quality of life (Al‐Sobayel et al., 2016; Bakalidou et al., 2013) have been reported. Divergent validity has been supported by the differences found between MS patients and healthy controls in the FSS (Armutlu et al., 2007; Bakalidou et al., 2013; Valko et al., 2008). The FSS has shown high internal consistency as analysed with Cronbach's alpha (Al‐Sobayel et al., 2016; Amtmann et al., 2012; Armutlu et al., 2007; Bakalidou et al., 2013; Lerdal et al., 2005; Ottonello et al., 2016; Valko et al., 2008) as well as high test‐retest reliability (Kleinman et al., 2000; Krupp et al., 1989; Learmonth et al., 2013; Rietberg et al., 2010).

As the validity and reliability of an assessment are contextual (i.e., related to the specific patient group studied) and cultural factors may affect the evaluation of fatigue, translated versions need to be validated. No validation studies of the FSS or other commonly used fatigue scales, like the Fatigue Scale for Motor and Cognitive Fatigue (FSMC) (Penner et al., 2009) or the Modified Fatigue Impact Scale (MFIS) (Multiple Sclerosis Council for Clinical Practice Guidelines, 1998), among patients with MS have been done for Finnish versions. The aim of this study was to evaluate the psychometric properties of the Finnish version of the FSS and to describe the results among patients with MS. The specific aims were to evaluate the validity and reliability of the FSS and its dimensional structure.

## 2 MATERIALS AND METHODS

### 2.1 Patients

This was a retrospective, cross‐sectional postal survey. The study protocol was approved by the ethics committee of the Hospital District of South‐Western Finland and all participants provided written informed consent. The study population included patients registered with the Finnish Neuro Society, a national patient association in Finland. The inclusion criteria comprised diagnosis of MS, age ≥18 years, a membership for at least 1 year, a permission to receive mail from the association, ability to complete the survey in the Finnish language, no other illness other than MS that could limit their participation, and no recent enrolment in a clinical trial. Recruitment letters were mailed to a random sample of 1,500 patients with MS (drawn by an independent statistician) from a pool of 5,408 patients with MS registered in the Finnish Neuro Society and fulfilling the eligibility criteria. Overall, 553 patients completed the questionnaire and were included in the analysis.

### 2.2 Outcome measures

Patients were required to complete the survey questionnaire or were interviewed via telephone using the Finnish questionnaire adapted from that used in previous, multi‐national studies (Karampampa, Gustavsson, & Miltenburger, 2013). The questionnaire included demographic background variables (e.g., age, gender, employment status, and early retirement due to MS), disease information (e.g., year of diagnosis, age at the diagnosis, type of MS, and self‐assessment of disease severity by Patient Assessment of Expanded Disability Status Scale (EDSS) Levels (a method widely used in cost‐of‐illness studies in MS (Kobelt, Berg, Lindgren, & Jönsson, 2006)). The self‐perceived feelings of fatigue were evaluated with the FSS (Krupp et al., 1989). The study population and methods have been described previously, (Ruutiainen, Viita, Hahl, Sundell, & Nissinen, 2016). The perceived quality of life was evaluated using the generic EuroQol 5D‐3L instrument (EQ‐5D) including five domains of well being (mobility, personal care, usual activities, pain/discomfort, and anxiety/depression) using a social tariff established with the general population in UK (EuroQol Group, 1990). The EQ‐5D has been officially translated into Finnish in 1991. The visual analog scale (VAS) was used to assess patients’ perceived health state on a scale of 0 (worst imaging health state) to 100 (best imaginable health state) (EuroQol Group, 1990). The physical and psychological impacts of the disease were assessed with the Multiple Sclerosis Impact Scale (MSIS‐29) (Hobart, Lamping, Fitzpatrick, Riazi, & Thompson, 2001). The MSIS‐29 is a 29‐item questionnaire structured in two subscales – a 20‐item scale for physical impairment and a nine‐item scale for psychological impairment. The Finnish version of the MSIS‐29 has been found to have satisfactory psychometric properties (Rosti‐Otajärvi, Hämäläinen, Wiksten, Hakkarainen, & Ruutiainen, 2017).

An authorized native Finnish speaking translator translated the questionnaire from US English into Finnish. The Finnish version was discussed twice with a group of two health professionals with experience in studying fatigue. Three pilot tests were performed. Two pilot tests were carried out to assess respondents’ understanding of both the items and the response categories of the FSS. After pilot testing, FSS was back‐translated by an authorized translator. The back‐translation was discussed by academic staff fluent in English. Dr L Krupp, who developed the original English version, approved the final back translation (Surakka, Romberg, Ruutiainen, & Virtanen, 2004; Surakka et al., 2004). Before the study started, the entire set of questions was subjected to a pilot test. Twenty individuals with MS who had filled out the questions were interviewed. Emphasis was placed on the respondents’ comprehension of each item. None of the individuals who participated in the pilot tests were part of the study population of this study. The items of the FSS are presented in English and Finnish in Table 1.

### 2.3 Statistical methods

Psychometric properties of the FSS were evaluated using standard methods (Nunnally & Bernstein, 1994) including:

- Data quality: percentage missing data and percentage computable scores
- Scaling assumptions: item mean scores, standard deviations (SDs), skewness, item to total correlations, and inter‐item correlations
- Acceptability: score range, mean scores, floor/ceiling effects, and skewness
- Reliability: Cronbach's alpha values with 95% confidence intervals, as well as Cronbach's alpha value when one item is deleted
- Validity: for evaluating construct validity of the FSS, Spearman correlation coefficients were used to examine the relationship between the FSS scores and burden of the disease (MSIS total, physical, and psychological scores), quality of life (EQ‐5D utility and VAS), as well as disease severity (Patient Assessment of EDSS Levels). Known‐group validity was determined by examining the FSS scores for subgroups of patients. Based on the previous literature, we predicted that (i) patients retired due to their MS would have scores higher than those still employed; (ii) patients with greater disease severity would have scores higher than those with milder disease severity; (iii) patients with progressive disease phenotype (secondary progressive or primary progressive) would have scores higher than those with relapsing‐remitting form of the disease; and instead (iv) patients of different age and gender would have similar scores. For comparisons between the two groups (gender, employment status), Student's *t* tests were used. In comparisons among three or more groups (disease phenotype, disease severity, and age groups), the analyses of variance (ANOVA) were used. The Tukey honest significance difference test was used for post hoc pairwise comparisons following ANOVAs.
- Unidimensionality: A confirmatory factor analysis (CFA) was used to evaluate the dimensional structure of the FSS. The comparative fit index (CFI) was calculated to evaluate the fit of the FSS in a unidimensional model.

## 3 RESULTS

### 3.1 Demographic and clinical characteristics of the sample

The study sample (*n *=* *553) was representative of all ages, MS phenotypes and levels of disability. Sample demographics and disease characteristics are summarized in Table 2. The mean (*SD*) age was 53.8 (11.4) years. A majority (76.1%) of the patients were within the working age (<63 years). The mean patient‐assessed EDDS score of the study sample was 4.0 (2.5).

**Table 2:** Sample demographics and disease characteristics (*n *=* *553)

Gender, *n* (%)	
Female	435 (78.7)
Age, years	
Mean (*SD*)	53.8 (11.4)
Range	21–88
Current employment situation, *n* (%)	
Employed or self‐employed	195 (35.3)
Student	2 (0.4)
Unemployed	23 (4.2)
On disability pension (any reason)	223 (40.3)
On retirement pension	110 (19.9)
Diagnosis	
Age at diagnosis, mean (*SD*)	37.4 (10.1)
Years since diagnosis, mean (*SD*)	16.4 (9.3)
Disease phenotype, *n* (%)	
Relapsing‐remitting	244 (44.1)
Primary progressive	94 (17.0)
Secondary progressive	160 (28.9)
Unknown	55 (10.0)
Disease severity	
EDDS score, mean (*SD*)	4.0 (2.5)

EDDS, Patient Assessment of Expanded Disability Status Scale Levels; *SD*, standard deviation.

### 3.2 Data quality

The percentage of missing data was low (0.4%), and the percentage computable scale scores were high (99.6%; Table 3).

**Table 3:** Data quality, scaling assumptions, acceptability, and reliability of the FSS

Psychometric property	FSS total
Data quality (*n* = 553)	
Subjects with missing items, *n* (%)	2 (0.4)
Number of missing items, *n* (%)	2 (<0.01)
Computable scale scores, *n* (%)	551 (99.6)
Scaling assumptions (*n* = 551)	
Item mean score, range	3.9–5.2
Item *SD*, range	1.8–2.2
Item skewness: range	−0.940–0.020
Item to total correlation, range	0.626–0.875
Item 1	0.626
Item 2	0.707
Item 3	0.846
Item 4	0.875
Item 5	0.854
Item 6	0.837
Item 7	0.866
Item 8	0.771
Item 9	0.795
Acceptability	
Possible score range	1–7
Observed score range	1–7
Score, mean (*SD*)	4.5 (1.7)
Floor, *n* (%)	14 (2.5)
Ceiling, *n* (%)	19 (3.5)
Skewness	−0.5
Reliability	
Cronbach's alpha (95% CI)	
Entire sample	0.949 (0.942–0.955)
Cronbach's alpha when one item deleted: range	0.939–0.951
Cronbach's alpha (95% CI)	
Age groups	
<40 years	0.950 (0.930–0.966)
40–49 years	0.941 (0.924–0.956)
50–59 years	0.951 (0.939–0.961)
60–69 years	0.959 (0.947–0.969)
≥70	0.942 (0.912–0.964)
Gender groups	
Female	0.950 (0.942–0.957)
Male	0.946 (0.930–0.960)
EDDS groups	
0–3	0.952 (0.943–0.961)
4–6.5	0.935 (0.922–0.947)
7–9	0.957 (0.942–0.970)
Disease phenotype groups	
Relapsing‐remitting (95% CI)	0.944 (0.932–0.954)
Secondary progressive (95% CI)	0.940 (0.925–0.953)
Primary progressive (95% CI)	0.957 (0.943–0.969)
Unknown disease phenotype (95% CI)	0.954 (0.934–0.971)

CI, confidence interval; FSS, Fatigue Severity Scale; *SD*, standard deviation.

### 3.3 Scaling assumptions

The frequency distribution of item response was relatively symmetrical; item mean scores ranged from 3.9 to 5.2 (*SD*, 1.8–2.2). Item to total correlations were acceptable (range, 0.626–0.875; Table 3). Additionally, all inter‐item correlations were strong (range, 0.424–1.00; Table 4).

**Table 4:** Inter‐item correlations for the FSS

	FSS 1	FSS 2	FSS 3	FSS 4	FSS 5	FSS 6	FSS 7	FSS 8	FSS 9
FSS 1	1.00								
FSS 2	0.424	1.00							
FSS 3	0.539	0.732	1.00						
FSS 4	0.596	0.685	0.809	1.00					
FSS 5	0.534	0.641	0.782	0.818	1.00				
FSS 6	0.520	0.688	0.747	0.782	0.777	1.00			
FSS 7	0.564	0.644	0.757	0.806	0.769	0.802	1.00		
FSS 8	0.570	0.523	0.652	0.670	0.662	0.650	0.707	1.00	
FSS 9	0.577	0.503	0.666	0.694	0.739	0.656	0.738	0.788	1.00

FSS, Fatigue Severity Scale.

### 3.4 Acceptability

Scale scores spanned the entire scale range and were not notably skewed; mean (*SD*) score of 4.5 (1.7) was relatively near the scale mid‐point, and floor and ceiling effects were negligible (2.5% and 3.5%, respectively; Table 3). In the total sample, 360 (65%), 307 (56%), and 265 (48%) patients were classified as fatigued when using a mean FSS cut‐off score of ≥4.0, ≥4.5, ≥5.0, respectively.

### 3.5 Reliability

Cronbach alpha reliability coefficient for the entire sample was 0.949 showing high degrees of internal consistency of the FSS. When deleting one item of the FSS, the Cronbach alpha values did not change markedly (range, 0.939–0.951; Table 3).

### 3.6 Validity

The correlations between the FSS and other outcomes are provided in Table 5. The construct validity of the FSS was confirmed by moderate/high Spearman's rank coefficient correlations between the FSS and burden of the disease (MSIS‐29), quality of life (EQ‐5D and VAS), and disease severity (EDDS). Higher fatigue scores were associated with a greater perceived burden of the disease, lower quality of life, and higher disease severity.

**Table 5:** Spearman correlations to assess construct validity of the FSS (*n *=* *553)

Construct	FSS
Burden of the disease (MSIS‐29 total score)	0.688
Physical burden of the disease (MSIS‐29 physical)	0.609
Psychological burden of the disease (MSIS‐29 psychological)	0.636
Quality of life (EQ‐5D utility)	−0.480
Quality of life (VAS)	−0.508
Severity of the disease (EDDS)	0.335

EDDS, Patient Assessment of Expanded Disability Status Scale Levels; EQ‐5D, EuroQol 5D‐3L instrument; FSS, Fatigue Severity Scale; MSIS‐29, Multiple Sclerosis Impact Scale; VAS, visual analog scale.

Known‐group validity was also supported (Table 6). As predicted, mean fatigue scores for patients who were retired due to their MS were significantly higher than that for patients who were employed, when limiting the comparison to age groups <63 years. Additionally, mean fatigue scores for patients with greater disease severity were higher than that for patients with milder disease severity. Similarly, mean fatigue score for patients with progressive disease phenotype (secondary or primary progressive) was higher than that for patients with relapsing‐remitting form of the disease. In contrast, mean fatigue scores did not differ according to age groups or gender.

**Table 6:** FSS group differences

Variable	FSS total, mean (*SD*)
Age, years	
<40 (*n *=* *70)	4.1 (1.8)
40–49 (*n *=* *123)	4.5 (1.7)
50–59 (*n *=* *184)	4.4 (1.7)
60–69 (*n *=* *130)	4.7 (1.8)
≥70 (*n *=* *44)	4.3 (1.7)
Mean difference (*F* test *p*‐value)	.216
Gender	
Female (*n *=* *433)	4.5 (1.7)
Male (*n *=* *118)	4.4 (1.7)
Mean difference (*t* test *p*‐value)	.790
Employment status (All subjects)	
Employed or self‐employed^1^ (*n *=* *194)	3.9 (1.7)
Student^2^ (*n *=* *2)	3.4 (1.3)
Unemployed^3^ (*n *=* *23)	4.6 (1.8)
On disability pension^4^ (*n *=* *222)	4.9 (1.6)
On retirement pension^5^ (*n *=* *110)	4.5 (1.8)
Mean difference (*F* test *p*‐value)	<.0001^1≠4,5^
Employment status (subjects aged <63 years, *n *=* *419)	
Disability pension due to MS (subjects aged <63 years; *n *=* *198)	4.9 (1.6)
All other subjects aged <63 years (*n *=* *221)	4.0 (1.7)
Mean difference (*t* test *p*‐value)	<.0001
EDDS	
0–3^1^ (*n *=* *243)	3.9 (1.7)
4–6.5^2^ (*n *=* *227)	5.0 (1.5)
7–9^3^ (*n *=* *81)	4.6 (1.9)
Mean difference (*F* test *p*‐value)	<.0001^1≠2,3^
Disease phenotype	
Relapsing‐remitting^1^ (*n *=* *243)	4.1 (1.7)
Secondary progressive^2^ (*n *=* *160)	5.0 (1.5)
Primary progressive^3^ (*n *=* *94)	4.9 (1.7)
Unknown (*n *=* *54)^4^	3.8 (2.0)
Mean difference (*F* test *p*‐value)	<.0001^1≠2,3; 2≠4; 3≠4^

EDDS, Patient Assessment of Expanded Disability Status Scale Levels; FSS, Fatigue Severity Scale; MS, multiple sclerosis; *SD*, standard deviation. Superscript numbers refers to subgroups and differences between them.

### 3.7 Dimensionality

The CFA results supported the unidimensionality of the FSS. The CFI for the FSS was 0.938 (χ^2^ **=* *309.0331, *df *=* *27).

## 4 DISCUSSION

This study examined the psychometric properties of the FSS in a large sample of Finnish patients with MS. Consistent with the findings from other language versions of the FSS (Al‐Sobayel et al., 2016; Amtmann et al., 2012; Armutlu et al., 2007; Bakalidou et al., 2013; Krupp et al., 1989; Learmonth et al., 2013; Lerdal, Johansson, Kottorp, & von Koch, 2010; Valko et al., 2008), the present study demonstrated satisfactory psychometric properties for the Finnish version according to classical test theory.

The data quality of the FSS was excellent, with 99.6% of computable scale scores. The mean (*SD*) FSS score in this study (4.5 [1.7]) was in line with English (4.8 [1.3] (Krupp et al., 1989); 5.1 [1.5] (Amtmann et al., 2012)), Greek (4.4 [1.8] (Bakalidou et al., 2013)), Turkish (4.8 [1.5] (Armutlu et al., 2007)), and Swiss (4.7 [1.6] (Valko et al., 2008)) versions, showing that the influences of language and cultural background might not be significant in the FSS among patients with MS. In the total sample, 360 (65%) patients were classified as fatigued when using a score of ≥4.0 as a criterion for self‐perceived fatigue. When using more stringent scores (≥4.5) 56% and (≥5.0) 48% of the patients in the present sample were evaluated as fatigued. Using a score of ≥4.0 as a criterion for possible fatigue is supported by the overall frequency estimates (80%) (Minden et al., 2006; Weiland et al., 2015). Typically floor and ceiling effects are considered problematic when more than 15% of the sample has either the lowest or the highest possible score (Terwee et al., 2007). In our study sample, the FSS did not show ceiling (3.5%) or floor (2.5%) effects of this magnitude supporting previous findings (Amtmann et al., 2012).

The reliability analyses included estimation of item to total correlations and internal consistency. High item to total correlations (*r*, range 0.626–0.875) provide evidence of item homogeneity for the FSS. In the present MS sample, the Finnish version of the FSS showed an excellent Cronbach's alpha of 0.95. Cronbach's alpha values did not differ significantly when one item of the FSS (range, 0.939–0.961) was deleted in a stepwise manner. Our results are in line with the previously reported high Cronbach's alpha values for the FSS among patients with MS, which have varied from 0.84 to 0.95 (Al‐Sobayel et al., 2016; Amtmann et al., 2012; Armutlu et al., 2007; Bakalidou et al., 2013; Ottonello et al., 2016; Valko et al., 2008). The optimal Cronbach's alpha range has been reported to be between 0.7 and 0.9 for internal consistency or item homogeneity, while values over 0.9 have been suggested to show item redundancy (Boyle, 1991). Our results together with previous findings suggest some redundancy in item content in the FSS and therefore a possibility to shorten the scale without a significant loss of precision. Item numbers 1 and 2 have previously shown relatively low inter‐item correlations (Lerdal et al., 2005) and reliability (Bakalidou et al., 2013). Subsequently, based on Rasch models, it has been suggested that by eliminating item number 1 (Ottonello et al., 2016), or item numbers 1 and 2 (Lerdal et al., 2010), better psychometric properties than those in the original nine‐item version may be obtained. Based on the Rasch analyses, even a shorter five‐item version (by eliminating item numbers 1, 2, 6, and 8) that satisfies strict tests of unidimensionality has been recommended (Mills, Young, Nicholas, Pallant, & Tennant, 2009). These shortened versions have however been found to show relatively high ceiling effects (Mills et al., 2009; Ottonello et al., 2016). Additionally, the five‐item version was found to be less sensitive to detect differences between groups and change over time (Lerdal et al., 2010). We found that inter‐item correlations and item to total correlations were the lowest for the item numbers 1 and 2 (item to total *r*, 0.626 and 0.707, respectively). However, these correlations were also considerably higher than the 0.40 threshold value that is commonly interpreted as an evidence of scale reliability (Everitt, 2002). Additionally, in CFA, the FSS showed a CFI of 0.94. A CFI of ≥0.90 has been suggested as a criterion for acceptable fit of the scale in a unidimensional model (Hu & Bentler, 1999). Previously reported CFIs for the FSS were 0.97 (Amtmann et al., 2012) and 0.99 (Bakalidou et al., 2013). These findings are well above the recommended threshold and support unidimensionality of the FSS. Moreover, in other studies using CFA the unidimensionality of the FSS has been supported (Al‐Sobayel et al., 2016; Bakalidou et al., 2013). Taken together, our results show, that the Finnish version of the FSS can be used as an original nine‐item version.

Correlations with other health‐related measures and variables provided the evidence for the construct validity of the FSS. The direction, magnitude, and pattern of correlations were consistent with predictions. Moderate/high correlations were found between the FSS score and perceived burden of the disease, quality of life, and disease severity. In previous studies the FSS has shown significant correlations with physical MS symptoms (Learmonth et al., 2013), disease severity (Armutlu et al., 2007; Flachenecker et al., 2002; Valko et al., 2008), depression (Armutlu et al., 2007; Bakalidou et al., 2013; Flachenecker et al., 2002), pain (Amtmann et al., 2012), and quality of life (Al‐Sobayel et al., 2016; Bakalidou et al., 2013). The results also confirmed the hypothesized group differences based on previous findings concerning employment status (Johansson et al., 2008; Mills & Young, 2010), disability (Amtmann et al., 2012; Armutlu et al., 2007; Mills & Young, 2010; Valko et al., 2008), disease phenotype (Mills & Young, 2010), as well as the demographic factors, age and gender (Valko et al., 2008). Progressive disease (higher disability and progressive phenotype) as well as retirement due to MS were found to be associated with higher levels of fatigue as evaluated by the FSS. In contrast, age or gender did not have an effect on the FSS scores.

The limitations of this study should be considered. The response rate was relatively low (37%) (Ruutiainen et al., 2016). Thus, it is possible that the sample is not representative. The responders can be argued to have more severe fatigue than the non‐responders which may increase the risk for “selection bias.” As described previously (Ruutiainen et al., 2016), the demographic and disease related characteristics of the study population represent well the general MS population. Additionally, the evaluations, including the severity and the phenotype of the disease, were based on patients’ self‐reports. Although this method is widely used in cost‐of‐illness studies in MS (Kobelt et al., 2006), we cannot rule out the possibility that some of the evaluations might have been different if based on clinician's evaluation. Possible “selection bias” and “information bias” may affect the generalisability of the findings of this study. Further, since depressive patients were not excluded from the study sample and depression was not evaluated, we cannot rule out the effects of depressive symptoms on the FSS scores observed in this study. Cross‐sectional data did not allow evaluation of the responsiveness of the FSS for change, an important aspect of psychometric functioning. The evaluation of test‐retest reliability or comparison of the FSS to other fatigue scales or between MS patients and healthy controls was not possible. Traditional methods comparable with previous studies were adopted to establish reliability and validity in this study. Strengths of the study include good data quality at least partly explained by the possibility to fill in the questionnaires via telephone interview.

In conclusion, this study supported the validity and reliability of the Finnish version of the FSS in patients with MS. The scale appears psychometrically feasible to assess perceived fatigue among Finnish patients with MS.

## CONFLICT OF INTEREST

None related to this specific study.
